# Role of GD3 Synthase ST8Sia I in Cancers

**DOI:** 10.3390/cancers14051299

**Published:** 2022-03-03

**Authors:** Angelina Kasprowicz, Groux-Degroote Sophie, Chann Lagadec, Philippe Delannoy

**Affiliations:** 1 University of Lille, CNRS, UMR 8576-UGSF-Unité de Glycosylation Structurale et Fonctionnelle, F-59000 Lille, France; angelina.kasprowicz@univ-lille.fr; 2 University of Lille, CNRS, Inserm, CHU Lille UMR9020-U1277-CANTHER Cancer Heterogeneity, Plasticity and Resistance to Therapies, F-59000 Lille, France; chann.lagadec@inserm.fr

**Keywords:** ganglioside, GD3 synthase, epithelial–mesenchymal transition, transcriptional regulation

## Abstract

**Simple Summary:**

The carbohydrate moiety of cell surface glycolipids is modified in cancers of neuro–ectoderm origin, leading to the expression of more complex structures with two or more sialic acid residues. These alterations result from the upregulation of the *ST8SIA1* gene that encodes GD3 synthase, the enzyme controlling the biosynthesis of complex gangliosides, and are usually associated with a more aggressive phenotype and a poor outcome for patients, making GD3 synthase an interesting target for cancer therapy. This review reports our general knowledge of GD3 synthase gene expression and regulation, its role in both epithelial–mesenchymal transition (EMT) and cancer progression, and the different approaches targeting GD3S expression in cancers.

**Abstract:**

GD3 synthase controls the biosynthesis of complex gangliosides, bearing two or more sialic acid residues. Disialylated gangliosides GD3 and GD2 are tumor-associated carbohydrate antigens (TACA) in neuro–ectoderm-derived cancers, and are directly involved in cell malignant properties, i.e., migration, invasion, stemness, and epithelial–mesenchymal transition. Since GD3 and GD2 levels are directly linked to GD3 synthase expression and activity, targeting GD3 synthase appears to be a promising strategy through which to interfere with ganglioside-associated malignant properties. We review here the current knowledge on GD3 synthase expression and regulation in cancers, and the consequences of complex ganglioside expression on cancer cell signaling and properties, highlighting the relationships between GD3 synthase expression and epithelial–mesenchymal transition and stemness. Different strategies were used to modulate GD3 synthase expression in cancer cells in vitro and in animal models, such as inhibitors or siRNA/lncRNA, which efficiently reduced cancer cell malignant properties and the proportion of GD2 positive cancer stem cells, which are associated with high metastatic properties, resistance to therapy, and cancer relapse. These data show the relevance of targeting GD3 synthase in association with conventional therapies, to decrease the number of cancer stem cells in tumors.

## 1. Introduction

Changes in glycosylation is a common feature of cancer cells that affects both *N*- and *O*-glycosylproteins as well as glycophingolipids, leading to the expression of tumor-associated carbohydrate antigens (TACA). These changes in glycosylation are usually explained by changes in the expression of specific glycosyltransferases, and are associated with increased aggressiveness of the tumors and a poor prognosis for the patients [1]. Gangliosides constitute a subclass of glycophingolipids substituted by one or more sialic acid residues. In humans, sialic acid molecules are exclusively *N*-acetyl-neuraminic acids (Neu5Ac) that can be *O*-acetylated, mainly on C9 [2]. Gangliosides are essential compounds of the plasma membrane, notably expressed at the outer leaflet in microdomains named “glycosynapses”, where they interact with cholesterol, phospholipids, transmembrane receptors, and signal transducers, controlling carbohydrate-dependent cell adhesion and signaling [3]. Normal human tissues mainly express mono-sialyl gangliosides, such as GM3 or GM1a; alternatively, di- or tri-sialyl gangliosides, with two or three sialic acid residues linked to the Gal residue of lactosylceramide (LacCer-Gg_2_Cer), are essentially found in developing tissues, during embryogenesis, and are mainly restricted to the nervous system in healthy adults [4]. Within glycosynapses, gangliosides are important regulators of receptor tyrosine kinases (RTK), and therefore play major roles in cell proliferation, adhesion, and motility [5]. Basically, mono-sialyl gangliosides are usually considered as downregulators of RTK signaling, whereas di-sialyl gangliosides upregulate RTK activation and downstream signaling pathways [6]. In mammals, the expression of b- and c-series gangliosides increases under pathological conditions, including cancers [7]. Particularly, di-sialyl gangliosides GD3 and GD2 have been described as TACA in neuroectoderm-derived tumors, including melanoma, neuroblastoma, and glioblastoma, as well as in breast cancer [8]. Moreover, substantial evidence has demonstrated the implication of complex gangliosides in oncogenesis by mediating cell proliferation, migration, stemness, tumor growth, and angiogenesis, making di-sialyl gangliosides interesting therapeutic targets for cancer immunotherapy [9]. The overexpression of complex gangliosides in cancers is usually correlated with the upregulation of *ST8SIA1* gene expression, which encodes the key enzyme for complex ganglioside synthesis, GD3 synthase (CMP-*N*-acetylneuraminate, GM3 α2,8-sialyltransferase, or ST8Sia I, EC 2.4.99.8, GD3S). Despite the role of GD2 synthase (encoded by the *B4GALNT1* gene) as the enzyme directly responsible for GD2 synthesis, GD3S is the rate-limiting enzyme of GD2 biosynthesis in breast cancer stem cells that have undergone EMT. In breast cancer stem cells, GD2 is considered as the ganglioside responsible for cancer cell stemness and metastatic properties, and various immunotherapy strategies targeting GD2 are used, or in development, for numerous cancer types [10,11]. However, since GD2 synthesis depends on GD3S expression levels, targeting GD3S could be a valuable therapeutic approach, in combination with these conventional therapies. In this review, we summarize our current knowledge on GD3 synthase expression and regulation in cancers, its role in cancer progression and metastasis, as well as in EMT and stemness properties. Finally, we highlight the potential of GD3 synthase as a therapeutic target in cancer treatment, and describe the different strategies utilized for targeting GD3S expression.

## 2. Specificity of GD3 Synthase, the Enzyme That Controls the Biosynthesis of Gangliosides from b- and c-Series

The sialylation reaction is catalyzed by sialyltransferases (ST), a family of membrane-bound glycosyltransferases that all transfer a sialic acid residue from the activated donor CMP-Neu5Ac onto an acceptor, which can be a neutral sugar (Gal, GalNAc, GlcNAc) or another sialic acid residue [12]. The sialylation reaction takes place in the Golgi apparatus at the end of the biosynthesis process of glycoconjugates; thus, STs are usually located in the trans-Golgi apparatus or trans-Golgi network. Twenty different sialyltransferases have been identified in humans, and all belong to the CAZy (carbohydrate-active enzymes) family #29 [13]. They are all type II transmembrane proteins, sharing the same structural organization, i.e., a short NH_2_-terminal cytoplasmic tail, a 16–20 amino acid transmembrane domain, a stem region variable in length (from 20 to 200 amino acids), and a large catalytic domain. These different STs vary in acceptor substrate specificity and in the nature of the linkage to the acceptor, thus defining four families of enzymes: ST3Gal, ST6Gal, ST6GalNAc, and ST8Sia.

Six different human ST8Sia family members have been identified. They all transfer a sialic acid residue to another sialic acid of α2,8 linkage, participating in the biosynthesis of oligo- and/or polysialylated glycan chains [14]. According to the substrate specificity of ST8Sia enzymes, ST8Sia I (EC 2.4.99.8) is the unique human sialyltransferase that synthesizes GD3 (II^3^Neu5Ac_2_Gg_2_Cer). The enzyme uses CMP-sialic acid as donor substrate and GM3 (II^3^Neu5AcGg_2_Cer) as an acceptor, and catalyzes the transfer of a sialic acid residue in α2,8-linkage. ST8Sia I is highly expressed during the early stages of embryonic development [15,16], where it participates in cell differentiation and proliferation [17]. In adult human tissues, ST8Sia I is mainly expressed in the nervous system [18]. Moreover, ST8Sia I and its direct product GD3, as well as GD2 synthesized from GD3 by the action of β4GalNAcT-I, are overexpressed in different types of cancers, mainly of neuroectoderm origin [19,20,21,22].

Human ST8Sia I was isolated in 1994 by three different research groups [23,24,25]. ST8Sia I is a typical type II Golgi enzyme, composed of a 12 amino acids (aa), a cytoplasmic tail, a transmembrane domain of 20 aa, and a large catalytic domain that includes the conserved sequence motifs (sialyl motifs) involved in the binding of substrates and the transfer of sialic acid [12].

Despite the clear role of GM3 as the main acceptor substrate for ST8Sia I, it has been demonstrated that the human recombinant enzyme can also use GD3 to synthesize GT3 (II^3^Neu5Ac_3_Gg_2_Cer) [18]. In addition, MDA-MB-231 breast cancer clones overexpressing GD3S accumulate GD3 and GD2 (II^3^Neu5Ac_2_Gg_3_Cer), as well as GT3 and GT2 (II^3^Neu5Ac_3_Gg_3_Cer), at the cell surface [26]. Furthermore, it was shown that MCF-7 breast cancer cells overexpressing GD3S also synthesized unusual derivatives of LacCer, i.e., GQ3 (II^3^Neu5Ac_4_Gg_2_Cer) and GP3 (II^3^Neu5Ac_5_Gg_2_Cer) [27]. ST8Sia I can also use the gangliosides GM1b (IV^3^Neu5AcGg_4_Cer), GD1a (II^3^Neu5Ac, IV3Neu5AcGg4Cer), or GT1b (II^3^Neu5Ac_2_,IV^3^-Neu5AcGg_4_Cer) as acceptor substrates, although with much lower efficiency [28], ST8Sia V being the main enzyme with GT1a/GQ1b synthase activity [29]. ST8Sia I is, therefore, the only GD3 synthase (GD3S) controlling the biosynthesis of gangliosides from the b- and c-series.

## 3. *ST8SIA1* Gene Expression and Regulation in Cancers

The *ST8SIA1* gene encoding GD3S is localized on chromosome 12 (p12.1–p11.2 locus). It is composed of five coding exons, spanning over 135 kbp [30]. Understanding the transcriptional regulation of *ST8SIA1* is critical to decipher the mechanisms underlying increased GD3S activity in numerous cancers.

High *ST8SIA1* expression has been described in numerous cancers, and is associated with cancer aggressiveness and poor outcome for patients. Notably, *ST8SIA1* expression is increased in the neurospheres and human glioblastoma multiforme tissues, whereas the inhibition of *ST8SIA1* results in decreased glioblastoma stem cell-associated properties [31]. Lack of *ST8SIA1* expression resulted in the prolonged lifespan of glioma-bearing mice, and low-grade pathology in generated gliomas, by modulating the AP2/MMP9 axis [32]. In breast cancer, high *ST8SIA1* expression has been associated with poor prognosis and high histopathological grading [33]. In particular, high *ST8SIA1* expression was observed in patients with the basal-like breast cancer subtype (together with high *MET* oncogene expression), as well as in breast cancer stem cells (CSC), compared to non-CSCs, which exhibit lower motility and lower mammosphere formation [34,35].

**Figure 1:**
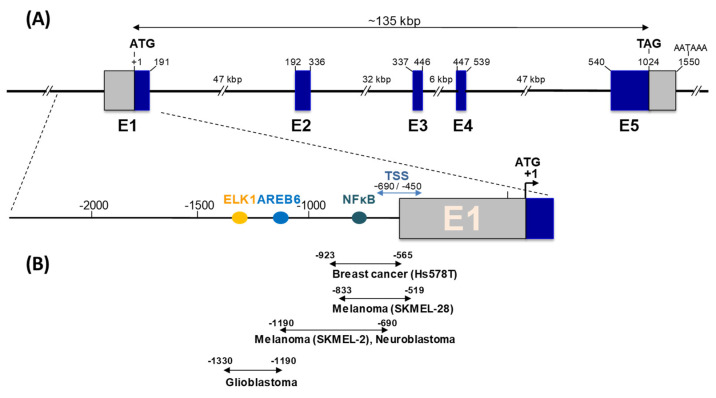
Human GD3 synthase gene (*ST8SIA1*) and promoter organization. (**A**) GD3 synthase gene organization. The coding region, represented by blue boxes, spreads over five exons (E1-E5). The noncoding regions are indicated by light gray boxes. (**B**) Schematic representation of the 5′-untranslated region upstream of exon 1. Transcription start sites (TSS) and identified binding sites for relevant transcription factors are indicated. Double-headed arrows show the minimal core promoters identified in the different cancer cell lines.

Consequently, the promoter region of the *ST8SIA1* gene has been studied in various cell types, such as melanoma [30,36], glioblastoma [37], neuroblastoma [38], and breast cancer cell lines [39]. A major transcript, T1, with transcription start sites (TSS) located 400 to 650 bp upstream of the initiation codon on the first exon, was described in most cell types. In breast cancer samples, two weakly expressed additional transcripts, T2 and T3, with different 5′-ends located upstream of the E2 exon, were also characterized using 5′-RACE experiments [39]. However, T1 is the major transcript in both Hs578T triple negative breast cancer cells and in breast tumors (Figure 1). Two different TSSs have been described in exon E1, leading to two transcripts differing in their 5′ ends, coding for protein isoforms of 356 and 341 aa, respectively [18,24]. The nucleotide sequence surrounding the second AUG (Met_16_) seems more favorable for translation initiation, regarding the model described by Kozak [40]. Moreover, data from Sasaki and co-workers suggest that the short *ST8SIA1* isoform, of 341 aa with a 12 aa cytoplasmic tail, is preferentially expressed in vivo [24].

In SK MEL-2 melanoma cells, the promoter region lacks TATA and CAAT boxes, but contains putative binding sites for general transcription factors associated with cancers and inflammation (c-Ets-1, CREB, AP-1, NF-κB), with a crucial role of NF-κB in *ST8SIA1* expression in SK-MEL-2 cells (Figure 1). Similarly, NF-κB is involved in the activation of *ST8SIA1* transcription in Fas-induced Jurkat T cells [41]. In U-87MG and T98G glioblastoma cells, the promoter region also lacks TATA and CAAT boxes, but contains binding sites for AREB6 and Elk-1 transcription factors, which are required for promoter activity [37]. In SK-N-BE(2)-C neuroblastoma cells, the *ST8SIA* promoter region also contains binding sites for c-Ets-1, CREB, and AP-1 transcription factors, and a critical NF-κB binding site that mediates valproic acid transcriptional activation [38]. In breast cancer cells, the core promoter of *ST8SIA1* contains two putative estrogen response elements (ERE) and a NF-κB binding site critical for promoter activity. In ERα positive breast cancer cells, estradiol represses *ST8SIA1* expression by inhibiting p65 and p50 nucleus localization and NF-κB binding to the *ST8SIA1* promoter (Figure 1) [39].

Additional transcriptional and epigenetic mechanisms involved in increased *ST8SIA1* expression have been studied. Firstly, in silico analysis suggested that the hypomethylation of *ST8SIA1* gene is linked to *ST8SIA1* overexpression in the most aggressive types of breast cancer cell lines and tissues tested [42]. Another mechanism of *ST8SIA1* regulation involves non-coding RNA (the non-coding RNA microRNA-33a (miR-33a) and has-let-7e (let-7e) can bind to the 3′-untranslated (3′-UTR) region of *ST8SIA1*), and their downregulation in colorectal cancer may contribute to higher *ST8SIA1* expression and increased cell motility and viability [43]. Long non-coding RNA (lncRNA) can also regulate *ST8SIA1* expression and cancer cell properties. In prostate cancer cells, *ST8SIA1* is a target of lncRNA MIR4435-2HG. The knockdown of lncRNA MIR4435-2HG expression inhibited prostate cancer cell malignant properties in vitro and in vivo, whereas the inhibition of *ST8SIA1* expression decreased the effects of miR4435-2HG, both in vitro and in vivo, by blocking the activation of the FAK/AKT/β-catenin pathway [44]. A better understanding of the multiple control levels of GD3S expression, the enzyme that controls GD3 and GD2 ganglioside expression, is key for defining new therapeutic strategies to decrease cancer cell malignant properties.

## 4. Role of GD3S in Cancer Progression and Metastasis

GD3S expression has long been associated with cancer progression and metastasis, especially in cancers of neuroectoderm origin; moreover, GD3 and GD2 are well-known melanoma- and neuroblastoma-associated antigens, respectively, playing key roles in cancer progression [8].

In terms of melanoma, the GD3 ganglioside has, for several decades, been known as a specific melanoma-associated carbohydrate antigen. Both GD3 and GD3S are absent in healthy human melanocytes, but show high expression in primary melanoma tissues, as well as in most melanoma cell lines, such as SK-Mel-28 [21,45,46]. Furthermore, highly metastatic cells show an increase in GD3S expression compared to poorly metastatic cells [47]. Inhibition of GD3S expression by antisense knockdown leads to a significant decrease in the expression of GD3 in hamster AbC-1 melanoma cells, and results in a marked decreased in tumor growth without affecting melanogenesis [48]. In addition, the stable transfection of GD3S cDNA in SK-Mel-28-N1 mutant cells that only express a-series gangliosides, leads to the conversion of GM3 into GD3, and the production of GD3S positive melanoma cells, which proliferate and migrate more than control cells [19,49]. These phenotypic changes are related to a high level of phosphorylation and activation of three major adaptor proteins: paxillin, p130Cas, and focal adhesion kinase (FAK) [49,50]. It was also shown that gangliosides, and the enzymes involved in their metabolism, are strictly interconnected with melanoma aggressiveness, and could represent a useful prognostic and diagnostic tool [51]. Moreover, the upregulation of GD3S and cell surface GD3 gangliosides was associated with human melanoma brain metastasis [52].

The expression of GTs implicated in ganglioside biosynthesis is also altered in brain tumors; therefore, the analysis of GT mRNA levels may be used for both diagnosis and prognosis. For example, a high expression of GD3S in glioma biopsies, together with a decrease in the expression of GM2/GD2 synthase, correlated with an increase in overall survival of patients [20].

The inhibition of the GD3 expression in rat F11 hybrid neuroblastoma cells by stable transfection with an antisense vector against the GD3S gene, was associated with reduced cell migration in vitro and reduced metastatic potential, in a nude mouse model [53]. In parallel, the overexpression of GD3S increases tumorigenicity and the invasion of rat glioma cells, whereas anti-GD3 mAb specifically inhibits tumor growth [54]. The stable transfection of GD3S cDNA into the U-251MG glioma cell line leads to the activation of Erk1/2, Akt, p130Cas, paxillin, and focal adhesion kinase signaling molecules, enhancing invasion activity, motility, and proliferation capacity in low or no serum concentrations without cell cycle arrest, which was achieved by avoiding the accumulation of p16 and p21 [55]. On the contrary, the lack of GD3S attenuated the malignant properties of gliomas in a genetically engineered mouse model [32].

Two clinical studies performed on public databases of tissues samples of invasive breast cancer have shown that GD3S displayed higher expression among ER-negative breast cancer tumors, and its overexpression was associated with poor pathohistological grading in ER-negative tumors [22,33]. A higher expression of *ST8SIA1* and *MET* was also observed in the basal-like subtype of human breast tumors [34]. The expression of GD3S is also known to be upregulated in osteolytic MDA-MET metastatic breast cancer cells [56].

GD3S expression in MDA-MB-231 cells induces the accumulation of b- and c-series gangliosides (GD3, GD2, and GT3) at the cell surface of MDA-MB-231 breast cancer cells, together with the acquisition of a proliferative phenotype under serum-free conditions [34,57]. GD3S expression increases the malignant properties of breast cancer cells by the specific and constitutive activation of the c-Met receptor by GD2, and the subsequent Erk/MAPK and PI3K/Akt signaling pathways [26].

## 5. Role of GD3S in EMT and Stemness Properties

Recent research into cancer cell heterogeneity has identified a small population of cancer cells, known as cancer stem cells (CSC), with high plasticity capability, self-renewal properties, and the ability to regenerate tumors when injected into immunodeficient mice over several generations. Furthermore, it has been shown that CSCs are the driving cell population in term of cancer progression and metastasis development [58]. Interestingly, this particular cell population is slow-cycling, and contains some quiescent cells that could account for chemotherapy and radiotherapy resistance [59]; these cells may also be associated with specific mechanisms, such as high ROS scavenger expression [60] or enhanced DNA damage responses [61]. Interestingly, the cell population can exit from dormancy and enter in an active cell cycle. Although typically symmetrical, CSC division gradually switches to differentiation or asymmetric divisions, allowing for the repopulation of the “damaged” tumor. Another effect hindering treatment is the ability of this population of CSCs to spread to distant organs through the lymphatic system or blood vessels. CSC able to reach into or rebuilt a new sustaining niche, could also reactivate an active cell cycle, growing to generate metastasis, which accounts for cancer relapse [59].

Importantly, both the establishment of a CSC-supportive tissue niche and the epithelial–mesenchymal transition (EMT) have been shown to be favored by many physiological phenomena. One of the conditions prone to this is an inflammatory environment. Indeed, transforming growth factor (TGF)-β1, tumor necrosis factor (TNF), or cytokines (interleukin (IL)-6) could all trigger EMT within differentiated tumoral hepatocytes, induced by a retro-differentiation program processing cells with a CSC phenotype [62,63]. Similarly, secreted IL-6 participates in EMT initiation in breast carcinoma. During the acquisition of a mesenchymal phenotype and invasive properties, cells re-express CD44, which has also been associated with CSC-like subpopulation enrichment [64,65]. In this context, GD3S expression has been associated with EMT marker expression, promoting cell migration, cell adherence/adhesion, and colony formation, reflecting an increase in malignant properties [66]. In parallel, GD3S can induce the expression of CSC markers, such as aldehyde dehydrogenase (ALDH) activity, and enhance the functional CSC property into mammosphere formation capability [67].

It has been demonstrated that the CSC population, defined as CD44^hi^/CD24^lo^, from human breast cancer patient samples and cell lines specifically express GD2, and could be used as a specific cell surface marker [67]. Indeed, *ST8SIA1* knockdown, reducing GD2/3 expression, induces the differentiation of CSCs towards a non-CSC phenotype, which could be highlighted by a functional assay as decreased formation. Most importantly, *ST8SIA1* knockdown annihilated tumorigenicity in an immunodeficient mice model, preventing tumor formation [35,66]. Furthermore, increased expression of GD3, induced by the constitutive activation of the c-Met signaling pathway, led to the enhancement of stem cell properties and an increase in metastatic potential [34]. In addition, GD3 and the EGF receptor have be observed to be colocalized in breast cancer stem cells; in this condition, GD3 participates to EGFR signaling activation. Interestingly, knocking down GD3S in MDA-MB-468 mammary carcinoma cells induces increased sensitivity, in vitro and in vivo, of the EGFR inhibitor gefitinib [67].

Yeh et al., highlighted that the GD3 ganglioside is overexpressed in neurospheres enriched with gastric cancer stem cell (GCSC) markers [31]. In an immuno-deficient mice model, sorted CD133+/GD3+ cells exhibited CSC properties with higher self-renewal potential, higher stemness gene expression panel (e.g., Nestin and Sox2), and, most importantly, higher tumorigenicity. Regarding GD3 synthase (GD3S) expression, Yeh et al., also found that GD3 ganglioside levels increased in neurospheres and human glioblastoma samples, but not in samples of normal brain tissues. Similar to the breast cancer model, stem cell-associated properties in glioma are affected by the inhibition of GD3S expression. Therefore, the use of neutralizing antibody targeting GD3 in vivo has been shown to induce cytotoxicity against GD3+ cells, which has a significant consequence on GBM tumor growth inhibition. Meanwhile, Woo et al., were able to validate the specific overexpression GD2 in glioma cancer stem cells compared to neural stem cells (NSC) [68]. Comparing GBM CSCs, with adult human NSCs as cell surface markers, Woo et al., confirmed the specific overexpression of GD2 in glioblastoma CSCs [68]. Nevertheless, evaluating GD2 as a specific CSC marker for cancer stemness, Woo et al., did not observed any difference in stemness properties between cells with or without GD2, using patient-derived glioblastoma primo-cultures. Both GD2+ and GD2- had similar functional characteristics to CSCs, shown using sphere formation capacity assay. Therefore, GD2 might not be useful as a therapeutic strategy to specifically target CSCs in glioblastoma.

In parallel, GD3S and GD2 expression are both significantly increased after the induction of EMT in transformed human mammary epithelial cells. On the other hand, GD3S inhibition, within mesenchymal breast cancer cells, disturbed EMT maintenance and prevented metastasis [69]. GD3S pharmaceutical inhibition, using triptolide (a small molecule specifically targeting GD3S), or transcription inhibition, using shRNA, prevents EMT initiation and maintenance initiated through Snail, Twist, and TGF-β1 signaling pathways; this alters the mesenchymal properties of SUM159 and MDA-MB-231, two claudin-low breast cancer cell lines. Interestingly, FOXC2, a key transcription factor for mediating several EMT pathways, binds to the GD3S promoter, regulating its expression. Therefore, the expression of GD3S drives EMT in cells, which maintain it through a retro-positive feedback loop via FOXC2 activation of the GD3S promoter. Finally, GD3S expression has been shown to correlate with adverse prognosis in patients with triple negative human breast tumors, known to be enriched in CSCs and mesenchymal cells.

## 6. Use of Inhibitors or Other Strategies Targeting GD3S Expression in Cancers

Considering that GD3S and its ganglioside products, mostly GD3 and GD2, have been clearly implicated in cancer cell properties, including migration, invasion, metastasis, stemness, and EMT transition, targeting GD3S appears as an attractive treatment strategy for GD3/GD2 positive cancers [70]. A first strategy could be to inhibit GD3S expression at the transcriptional level. As already mentioned in paragraph three, it has been shown in different cancer cell lines that the core promoter of GD3S contains binding sites for transcription factors, such as NF-κB [39,70,71]. In ER+ breast cancer cells, Bobowski et al., demonstrated that estradiol inhibits NF-κB-mediated activation by inhibition of p50/ p65 NF-κB subunit translocation into the nucleus, resulting in decreased GD3S expression [39]. As GD3S expression is regulated by NF-κB, interfering with the NF-κB pathway seems to be an effective strategy for decreasing cell malignant properties.

For that purpose, different NF-κB inhibitors, including triptolide, were used to influence GD3S expression. Triptolide was isolated from a Chinese medicinal herb that was used to treat inflammation, autoimmune diseases, and cancers. Sarkar et al., showed that triptolide induced an epithelial-like morphology in breast cancer cells [70]. Kwon et al., demonstrated that melanoma cells treated with triptolide showed decreased GD3S mRNA expression through the inhibition of NF-κB signaling [71]. It was further demonstrated that the target region for triptolide in the GD3S promoter is in fact the previously described region containing NF-κB binding sites [71].

Other inhibitors have been used, such as BMS-345541, an IKK inhibitor that also regulates NF-κB signaling. BMS-345541 induced a decrease in GD3S expression, inhibited the tumorigenic function of breast cancer stem cells in vitro, and inhibited tumor growth and metastasis in immunodeficient mice, by decreasing GD2 synthesis [72]. Therefore, BMS-345541 could be used to treat metastatic triple negative breast cancer cells in combination with conventional chemotherapy. A combination of different strategies was also used to further decrease cancer cell malignant properties. For example, Liang and coworkers used a combination of gefitinib, an EGFR inhibitor, and GD3S knockdown by shRNA to treat mice infected with breast cancer cells, and observed a suppression of tumor growth [67].

Over the last two years, more papers have highlighted that GD3S has a critical importance in EMT and tumor progression, and new mechanisms involved in the regulation of GD3S expression have been described, such as the role of long non-coding RNA. In vivo analyses of glioma in GD3S-KO mice models showed a decreased in the expression of genes implicated in cell cycle, extracellular matrix, and growth factor [32,73]. The long non-coding (lnc) RNA MIR4435-2HG that binds to the *ST8SIA1* gene is highly expressed in several cancers (lung, bladder, liver, prostate, stomach) and activates the FAK/AKT/B-catenin pathway in prostate cancer cells; furthermore, interfering with either *ST8SIA1* or MIR4435-2HG expression inhibited the malignant properties of cells both in vitro and in vivo [44]. Furthermore, Wan et al., demonstrated that GD3S protein levels were increased in chemoresistant triple negative breast cancer patients after chemotherapy. The inhibition of GD3S by siRNA decreased the viability of resistant cells vis the FAK/AKT/mTOR pathway [74]. These results suggest that SiRNA/lncRNA and/or SiRNA/lncRNA are expression vectors that regulate GD3S expression and potentially other genes involved in cancer cell malignancy are also promising approaches that can be used to overcome GD3/GD2+ cancers.

## 7. Perspectives

In the last few years, great advances have been made regarding the understanding of complex ganglioside biosynthesis, and their crucial roles in cancer cell malignant properties. GD3S is the key enzyme that controls b-series ganglioside biosynthesis, especially GD3 and GD2, which are the most described tumor-associated gangliosides, involved in the growth, proliferation, invasion, stemness, and metastasis of neuro–ectoderm derived cancers. Although the GD3S promoter and its transcriptional regulation have been studied in a variety of cancer cell types, other levels of regulation of GD3S expression, such as epigenetic regulation or the importance of non-coding RNA (miRNA, lncRNA), are not yet deciphered. Consequently, the mechanisms of GD3S overexpression in cancers, resulting in increased GD3/GD2 biosynthesis, largely remain to be elucidated.

Nevertheless, the major roles of GD3S and its products, GD3 and GD2, in cancers highlights the interest for the development of GD3S inhibitors for cancer therapy. In this respect, the only described GD3S inhibitor, triptolide, a natural compound isolated from the Chinese herbal plant *Tripterygium wilfordii*, has shown promising therapeutic effects in breast cancer, pancreatic cancer, prostate cancer, and bladder cancer. The development of new GD3S inhibitors, which could be used in combination with conventional therapies, especially those targeting complex gangliosides (anti-GD2 mAb immunotherapies) or RTKs, is a promising concept for the treatment of the most aggressive GD3/GD2 positive cancers. Furthermore, because the inhibition of *ST8SIA1* expression, achieved by various strategies (Si/ShRNA, miRNA, lncRNA), results in decreased cancer cell malignancy, both in vitro and in vivo, investigating databases for non-coding RNAs that target *ST8SIA1* (and potentially other genes involved in cancer progression) is a promising approach for future therapies. This strategy requires a deep knowledge of the different levels of *ST8SIA1* expression and the networks of the genes that are co-regulated in cancers.

Finally, recent data have highlighted a role for a special class of gangliosides, O-acetylated gangliosides, as cancer markers and therapeutic targets of interest [75]. The most studied O-acetyl-gangliosides are O-acetylated-GD3 (corresponding to GD3 with an O-acetyl group on the C9 of the terminal sialic acid residue) and O-acetylated-GD2. OAcGD2 was recently proposed as a cancer stem cell marker and a therapeutic target of interest in breast cancer [76]. The biosynthesis of these compounds depends on the expression and activity of GD3S and GD2S, but also on the sialate-O-acetyl-transferase (SOAT) CASD1 that adds an O-acetyl group onto a sialic acid residue carried by GD3 and GD2 gangliosides [77]. The biosynthetic mechanisms of ganglioside O-acetylation remain unclear, and the regulation of CASD1 expression and activity has not been studied. However, high CASD1/OAcGD2 expression increases the migration and invasion of SUM159PT triple negative breast cancer cells, and OAcGD2 is a marker of breast cancer stem cells, which have the highest tumor-initiating capacity [78]. Consequently, targeting OAcGD2 and CASD1 in cancers using immunotherapy, but also transcriptional or functional inhibitors of CASD1, might be another strategy used to decrease the malignant properties of cancer cells.
